# Acupuncture for the Treatment of Itch: Peripheral and Central Mechanisms

**DOI:** 10.3389/fnins.2021.786892

**Published:** 2022-03-30

**Authors:** Yi Tang, Shirui Cheng, Jin Wang, Yin Jin, Haodong Yang, Qihui Lin, Sanmei Xu, Lin Hui, Quanying Yin, Ying Yang, Xi Wu

**Affiliations:** Graduate School, Chengdu University of Traditional Chinese Medicine, Chengdu, China

**Keywords:** itch (pruritus), neurobiologic mechanisms, review, periphery and center, acupuncture-therapy

## Abstract

Despite the widespread clinical use of acupuncture in the treatment of pruritus caused by psoriasis, urticaria, uremic, and other diseases, insights into the mechanism of action of acupuncture are still emerging. For the above reasons, a beneficial effect of acupuncture on pruritus was not recommended or reported in recent clinical practice guidelines. Acupuncture is a kind of physical stimulation, which has the characteristics of multi-channel and multi-target effects. The biomechanical stimulation signal of acupuncture needling can be transformed into bioelectric and chemical signals; interfere with kinds of cells and nerve fibers in the skin and muscle; alter signaling pathways and transcriptional activity of cells, mediators, and receptors; and result in inhibition of peripheral and central transmission of pruritus. Available mechanistic data give insights into the biological regulation potency of acupuncture for pruritus and provide a basis for more in-depth and comprehensive mechanism research.

## Introduction

Pruritus is a sensation that provokes the desire to scratch (2019European S2k Guideline), which remains one of the most agonizing symptoms for affected patients and a clinical challenge for physicians (Weisshaar et al., 2019). Pruritus may be a result of dermatological diseases or systemic diseases (Satoh et al., 2021). The onset of pruritus with underlying dermatological disease usually coincides with the onset of typical skin lesions (Ständer et al., 2015). Pruritus caused by underlying systemic disorder, such as end-stage renal disease, hemodialysis, or primary biliary cirrhosis, is usually persistent or paroxysmal, and the peak itch sensation mainly occurs at night or in the evening (Millington et al., 2018; Satoh et al., 2021). Region, ethnicity, age, underlying diseases, methods of investigation, and access to the regional healthcare system influence the occurrence of pruritus (Weisshaar and Dalgar, 2009), which may explain the reason for the inconsistency of data reported by various countries or regions (Weisshaar et al., 2006; Weisshaar, 2016). A recent European multi-center study found that the incidence of itching in dermatological diseases was 54.5%, while the incidence in healthy people was 8% (Schut et al., 2019). In China, an epidemiological survey showed that 47.8% of patients with dermatological diseases were accompanied by itching symptoms (Zhang, 2014).

In the clinical practice, although the newly developed drugs for various causes have made great progress, it is also difficult to control the clinical symptoms of itching because the underlying biologic mechanism are not confirmed until now. Antihistamines are the most widely used systemic antipruritic drugs in dermatological diseases. They achieve the purpose of antipruritic by selectively blocking histamine H_1_ receptors and antagonizing the effects of histamine (Leslie et al., 2015; Thurmond et al., 2015). Long-term use of antihistamines not only cannot fundamentally solve the chronic pruritus (CP) in older Asian adults, but also increases their risk of falling (Moosa et al., 2021). Antihistamines are widely used for the treatment of CP associated with various systemic diseases. However, conventional doses of antihistamines in the treatment of pruritus in internal diseases have not proven to be effective (O’Donoghue and Tharp, 2005). About 30–90% of patients suffering from CP with cholestasis are unresponsive to antihistamines (Langedijk et al., 2021). Regarding non-histaminergic drugs, there are different characteristics in different drugs treating different diseases. Systemic glucocorticoids (GCs) are commonly used as a short-term treatment in severe CP associated with dermatological disease or systemic disease due to its serious side effects (Weisshaar et al., 2019). Cholestyramine, the first-line guideline-recommended treatment for cholestatic pruritus (Hirschfield et al., 2017), has a poor clinical effect on patients with excessive pruritus intensity (Düll and Kremer, 2020). Antidepressants are effective particularly in refractory CP, especially in malignant, cholestatic, and chronic kidney disease. However, side effects of antidepressants are common and include drowsiness, fatigue, and headache (Kouwenhoven et al., 2017).

Chronic pruritus has considerable, severe, and detrimental implications on the quality of life because it is difficult to heal (Whang et al., 2021a,b). Several studies by Gil, Yosipovitch and his colleagues found that pruritus seriously plays a great impact on people’s quality of life, including emotions, attention, eating habits, sexual function, and sleep (Yosipovitch et al., 2001, 2010, 2015). The longer the duration and severity of itching, the greater its substantially negative impact on work productivity and day-to-day activities (Chrostowska-Plak et al., 2012; Yano et al., 2013; Carr et al., 2014). Some patients with CP will lead to psychosocial comorbidities, including anxiety, depression, and even suicidal tendencies (Yosipovitch and Bernhard, 2013; Reszke and Szepietowski, 2018; Brenaut et al., 2019; Whang et al., 2020; Hawro et al., 2021; Umehara et al., 2021).

## Acupuncture

### The Clinical Effect of Acupuncture

As an important enabling part of Chinese medicine, acupuncture has been used in China for more than 2,500 years in the treatment of itch (van den Berg-Wolf and Burgoon, 2017). Acupuncture is a safe and effective therapy in the treatment of pruritus (Yao et al., 2016). Compared with sham acupuncture, verum acupuncture can significantly reduce the total SCORing Atopic Dermatitis index (SCORAD) (Kang et al., 2018; Park J. G. et al., 2021) and the visual analogue scale (VAS) for itching in patients with atopic dermatitis (Pfab et al., 2012), atopic eczema (Pfab et al., 2010), and hemodialysis (Nahidi et al., 2018). There are three RCTs on uremic pruritus that indicated that acupuncture (Che-Yi et al., 2005), acupressure or transcutaneous electrical acupoint stimulation (TEAS), and auricular acupressure can reduce the VAS for itching in patients with uremic pruritus (Yan et al., 2015; Kılıç Akça and Taşcı, 2016). Che-Yi et al. (2005) have found a significant reduction in pruritus scores of hemodialysis patients with refractory uremic pruritus during the 3-month follow-up after acupuncture. Coincidentally, the relief of pruritus after 4 weeks of acupuncture treatment of patients with mild to moderate atopic dermatitis has been observed (Park J. G. et al., 2021). According to Tables 1, 2, the most commonly used style of acupuncture was manual acupuncture, followed by intradermal acupuncture and acupressure. Electroacupuncture (EA), TEAS, and auricular acupuncture were only used in one study. Antipruritic points included Quchi (LI11), Zusanli (ST36), Xuehai (SP10), and Shaohai (HT3). There are eight RCTs studies that have used Quchi point, which has the functions of dispelling wind to releasing exterior, clearing heat and relieving itching.

**TABLE 1:** Effects of acupuncture treatment for itching.

Source	Country	Study design	Patient population	Sample Size (T/C), Dropout (T/C)	Treatment	Control	Primary outcome	Primary outcome result
Park J. G. et al., 2021	South Korea	A randomized, participant and assessor-blinded, sham-controlled trial, 2-arm	Atopic dermatitis	36(18/18), 1(18/17)	VA	SA	SCORAD (Total)	The mean difference was −11.83 (7.05) in the VA group and 0.45 (7.77) in the SA group (*P* < 0.0001)
Kang et al., 2018	South Korea	A randomized, controlled, single-blinded study, 3-arm	Atopic dermatitis	30(10/10/10),0	VA1/VA2	SA	SCORAD (Total)	A significant difference among the three groups at week 8 (*P* = 0.020), especially in the VA1 group compared to the SA group (*P* = 0.026).
Pfab et al., 2012	Germany	Patient - and examiner-blinded, randomized, placebo-controlled, crossover, 7-arm	Atopic dermatitis	20,1	VAp/VAa	PAp/PAa/AC/PC/NI	VAS for mean itch intensity	Mean itch intensity (SE: 0.31 each) was significantly lower following VAa (31.9) compared with all other groups (PAa: 36.5; VC: 36.8; VAp: 37.6; PC:39.8; PAp: 39.9; NI: 45.7; *P* < 0.05)
Lee et al., 2012	United States	An unblinded pilot RCT, 2-arm	Atopic dermatitis	15(8/7),3(1/2)	Acupressure with standard of care	Standard of care	VAS (Pruritus), IGA, EASI	A greater change in the acupressure group than in the control group in VAS score (*P* = 0.04), IGA (*P* = 0.03) and EASI lichenification score (*P* = 0.03)
Pfab et al., 2010	Germany	A double-blind (patient and observer), randomized, prospective, three-arm crossover trial	Atopic eczema	30,0	VA	PA/NA	VAS (Pruritus)	Drect effect: 35.7 ± 6.4 vs 40.4 ± 5.8 vs 45.9 ± 7.8,VA and NA(*P* = 0.009), VA and PA (*P* = 0.022); Preventive effect: 34.3 ± 7.1 vs 37.8 ± 5.6 vs 44.6 ± 6.2, VA and NA (*P* < 0.001),PA and NA (*P* = 0.002)
Nahidi et al., 2018	Iran	A randomized, Sham-controlled 2-arm	Pruritus in Hemodialysis Patients	30(15/15), 4(15/11)	VA+gabapentin +usual treatment and care	SA+gabapentin +usual treatment and care	VAS (Pruritus)	Before: 9.87 ± 0.35 vs 9.45 ± 0.93, *P* = 0.18; After: 3.93 ± 2.85 vs 8.18 ± 1.40, *P* < 0.001
Kılıç Akça and Taşcı, 2016	Turkey	A randomized, controlled trial, 3-arm	Uremic pruritus	75(25/25/25), 1(25/24/25)	Acupressure/TEAS +Antihistamine tablets +usual treatment and care	Antihistamine tablets+usual treatment and care	VAS (Pruritus)	Before: 6.84±1.70 vs 7.37±1.31 vs 6.92±1.41, *F* = 0.918, *P* > 0.05; After: 3.36 ± 2.37 vs 3.12 ± 2.15 vs 5.08 ± 1.55, *F* = 6.672, *P* < 0.05; Test value (*P* value): *t* = 5.346 (<0.001) vs *t* = 7.936 (<0.001) vs *t* = 3.942 (<0.05); Acupressure vs TEAS VAS: MD = 0.23, *P* = 1.000; Acupressure vs Control VAS:MD = −1.72, *P* = 0.013; TEAS vs Control VAS:MD = −1.95, *P* = 0.004
Yan et al., 2015	China	A randomized controlled trial, 2-arm	Uremic pruritus	62(32/30),0	Auricular acupressure, tape with Vaccaria seeds	No auricular acupressure, tape without Vaccaria seeds,	VAS (Pruritus)	3.844 ± 1.687 vs 5.567 ± 2.285, *F* = 22.32, *P* < 0.0001
Che-Yi et al., 2005	China	A randomized, Sham-controlled study, 2-arm	Refractory uraemic pruritus	40(20/20),0	VA	SA	A pruritus score questionnaire	Before: 38.2 ± 4.8vs 38.5 ± 3.2; After: 17.3 ± 5.5 vs 37.5 ± 3.2; At 3 months: 16.5 ± 4.9 vs 36.5 ± 4.6 (*P* < 0.001)

*VA, verum acupuncture; SA, sham acupuncture; PA, placebo acupuncture; NA, no acupuncture; VAp, preventive verum acupuncture; VAa, abortive verum acupuncture; PAp, preventive placebo acupuncture; PAa, abortive placebo acupuncture; VC, verum cetirizine; PC, placebo cetirizine; NI, no-intervention control; SCORAD, SCORing Atopic Dermatitis index score; VAS, visual analogue scale; EASI, Eczema Area and Severity Index; TEAS, transcutaneous electrical acupoint stimulation; IGA, the Investigator’s Global Assessment.*

**TABLE 2:** Acupoints and methods for relieving itching.

Source	Acupoints	Style of acupuncture	Needle type	Depth of insertion	Response sought	Stimulation	Retention time	Course of treatment
Park J. G. et al., 2021	(1) Fixed points: LI11, ST36, PC6 (bilaterally)	MA	Disposable sterile stainless steel needles (0.25 mm × 40 mm, Dongbang Acupuncture Inc., Bundang, Seongnam, South Korea)	5–30 mm, perpendicular to skin surface	“de qi” sensation	Manual stimulation, needle rotation with thumb and index fngers for the frst 10–15 s	15 min	twice-weekly for 4 weeks
	(2) Optional points:up to 10 acupoints. ➀ST43, GB41 for gastric stufness or dyspepsia; ➁ LI2, GB41 for tenderness around ST25, diarrhea, or constipation; ➂ TE3, SI3 for fullness in the chest and hypochondrium; ➃ TE3, TE6 for lower abdominal pain and tenderness on CV17;➄ SI3,GB41 for lower abdominal pain and dry skin; ➅ SI2, BL66 for lower abdominal pain and heat in the upper body and cold in the lower body; ➆ LR3 and SP3 for pain in the hypogastric region with darkness of the sublingual collateral vessels (GB41, TE3, SI3 can be applied bilaterally)							
	LI11 (bilaterally), auricular Shenmen (contralaterally)	IA	A hypoallergic PTN (1.5 mm, 10 mm × 10 mm adhesive tape, Haeng Lim Seo Won Medical Co., South Korea)	1.5 mm, perpendicular to skin surface	None	Press PTNS at LI11 for more than 3 min when they feel severe itching	1–2 days until PTN falls of	
Kang et al., 2018	(1) Fixed points: LI11, ST36, PC6 (bilaterally)	MA	A sterilised stainless steel needle (0.25 mm × 40 mm, Dongbang Acupuncture Inc., Bundang, Sungnam, South Korea)	5–30 mm, perpendicular to skin surface	“de qi” sensation	Manual stimulation, needle rotation with thumb and index fingers for the first 10–15 s	15 min	VA1: thrice weekly for 4 weeks (total 12 times); VA2: twice weekly for 4 weeks (total eight times)
	(2) Optional points: ➀ ST43, GB41 for gastric stuffiness or dyspepsi; ➁ LI2 and GB41 for tenderness around ST25, diarrhoea, or constipation; ➂ TE3 and SI3 for fullness in the chest and hypochondrium; ➃ TE3 and TE6 for lower abdominal pain plus tenderness on the chest centre; ➄ SI3 and GB41 or SI2 and BL66 for lower abdominal pain, dry skin, or heat in the upper body and cold in the lower body, GB41, SI2, and BL66 were chosen; ➅ LR3 and SP3 for pain in hypogastric region with darkness of the sublingual collateral vessels (GB41, TE3, SI3 can be applied bilaterally according to the signs or symptoms of the patient)							
	LI11 (bilaterally), Shenmen (the contralateral side)	IA	A hypoallergenic PTN (1.5 mm, 10 mm × 10 mm adhesive tape, Haeng Lim Seo Won Medical Co., South Korea)	1.5 mm, perpendicular to skin surface	None	Participants will be educated to press PTNs for 3 s when they feel severe itch	1–2 days or until PTN falls of	
Pfab et al., 2012	VAp:LI11, HT3, ST34, SP10 (dominant side)	Electroacupuncture	Sterile stainless steel needles (0.25 mm × 40 mm)	20–30 mm	/	Electrical stimulatione, 100 Hz, 0.2 ms pulse width, constant-current AS Super 4 Han device (Schwa-medico GmbH, Ehringshausen, Germany), the intensity was set to moderately strong but not painful	20 min	Total experimental time was 20 min, which included 27 warm–cool cycles
	VAa:LI11, HT3 (dominant side)							
Lee et al., 2012	LI11	Acupressure	Using a 1.2 mm titanium acupellet (Lhasa OMS, Weymouth, MA, United States)	/	/	/	3 min	thrice weekly for 4 weeks
Pfab et al., 2010	LI11, SP10 (dominant side)	MA	Sterile stainless steel needles (0.25 mm × 40 mm)	20–30 mm	/	Manual stimulation, manipulated for a 5 s period	11 min	/
Nahidi et al., 2018	SP6, SP10, LV3, LI4, LI11	MA	Acupuncture	10–30 mm	heaviness, numbness, or soreness	A specialist of acupuncture inserted needles into acupoints by using his fingertips while applying consistent pressure on the correct acupoint with small rotational movements	30 min	/
Kılıç Akça and Taşcı, 2016	LI11	Acupressure	/	/	soreness, numbness, heaviness, swelling, and warmth	the therapist used her fingertips, applying a consistent pressure on the correct acupoint with small rotational movements. The action was done rapidly at the rate of two rotations per second. The force was maintained between 3 and 5 kg	6–10 min	Thrice weekly for 4 weeks
		TEAS	A portable, battery-powered TEAS unit was used (XFT-320; Shenzhen XFT Electronics Ltd, Guangdong, China), 5 and 10 Hz (dense-dispersed waveform)		/	The pen provides massage effects like thumb pressure and patting	3 min	
Yan et al., 2015	CO10, CO14, CO15, TF4, CO18, AT4 (unilaterally, alternating bilaterally)	Auricular acupressure	Tape with vaccaria seed (Runshi Trading, China)	/	The patients felt a tolerable soreness, numbness, and heat	Pressure was applied to each ear point for 1–2 min with appropriate finger force	1–2 min, 5–8 times a day, with one mandatory press before going to sleep every night. The tape was replaced every other day and removed every Sunday as a break day	Thrice weekly for six weeks
Che-Yi et al., 2005	LI11 (unilaterally)	MA	A 1-inch 34-gauge acupuncture needle	/	/	Manual stimulation	60 min	Thrice weekly for 1 month

*VAp, preventive verum acupuncture; VAa, abortive verum acupuncture; MA, manual acupuncture; IA, intradermal acupuncture.*

However, the underlying mechanism of these consequences remain largely unknown. An important question is that whether these observed effects share a common mode of action or result from a variety of distinct processes. This review summarizes the physiological basis of acupuncture treatment of pruritus, explores the peripheral and central mechanisms of acupuncture to relieve itching, and proposes existing challenges and future research directions.

### Physiological Basis of Acupuncture Relieving Itch

Through opography of the cadavers, it was found that most of the acupoints on the meridians are dominated by peripheral nerves (Wick et al., 2007). Acupuncture points in humans may be excitatory muscle/skin–nerve complexes with high-density nerve endings (Li et al., 2004). The acupoint tissues are rich in various forms of receptors, such as free nerve endings and cyst receptors in the dermis, muscle spindles in muscle tissue, and Rufini bodies near the joint capsule. According to the relationship between meridian acupoints and nerves, the commonly used antipruritic acupoints Quchi, Zusanli, Hegu, etc. (Zhang et al., 2019) are located at the muscle movement points, and the nerve endings on the body surface are particularly dense (Guo, 2016). Because of the appropriate stimulation produced by various forms of acupuncture techniques such as hand acupuncture, electroacupuncture, and transcutaneous electrical stimulation, acting on receptor cells, the external stimulation is transformed into transmembrane electrical signals through channel proteins or membrane-specific receptors with specific sensory structures. For chemically sensitive receptors, the transduction process is related to the release of chemical substances. Chemical substances activate different receptors through direct and indirect effects, depolarizing the receptors to produce afferent impulses. The sensory terminals of itch neurons terminate in the epidermis and form branched free nerve terminals, which contain numerous membrane receptors for various mediators (Zylka et al., 2005; Imamachi et al., 2009; Han et al., 2013).

## Mechanism of Acupuncture for Acute Itch

Acute itch is defined as the duration of pruritus ≤6 weeks according to a recommendation by the International Forum for the Study of Itch (IFSI). The acute attack of pruritus is a reaction that eliminates the harmful environment of our body, which plays a positive protective effect role in our survival (Wang et al., 2021). As claimed by currently known studies, after allergens, external irritants, or pruritogens interface with the skin, the keratinocytes and local immune cells recruit and release a great deal of chemical mediators (Pasparakis et al., 2014). Mast cells activate and release the elements of cytoplasmic granules. These mediators bind to the membrane receptors of homologous mediators in the itch-sensitive neuron endings (histaminergic neurons and non-histaminergic neurons) (Ikoma et al., 2006) located in the epidermis. Then, the itching signal is generated (Dong and Dong, 2018). The itching signal is transmitted to the dorsal root ganglion (DRG) at the dorsal horn of the spinal cord through the unmyelinated fiber C and a small portion of Aδ nerve fibers for preliminary processing. After that, the processed itching signal is transmitted to multiple brain areas and circuits through the spinothalamic tract for processing (Bourane et al., 2015; Cevikbas and Lerner, 2020). These distinct brain areas are thought to be involved in different aspects of itch signal processing (Chen and Sun, 2020). The primary and secondary somatosensory cortices contribute to the localization, intensity rating, and recognition of and attention to itch (Yosipovitch and Mochizuki, 2015). The midcingulate cortex is activated during itch induction or itch-related behaviors, for it is closely related to the premotor planning and process of affective-motivational in itch (Schneider et al., 2008). The putamen is significantly activated during scratching of itch. The reason is that it is a critical component of the striato-thalamo-cortical circuity related to motivational processing, habitual behavior, and action initiation, which is the basis of scratching (Graybiel, 2008).

An important peripheral target of acupuncture in relieving acute pruritus is 5-HT and receptor, which is an important histamine-independent pruritus mediator (Hachisuka et al., 2010; Bautista et al., 2014). 5-HT can directly activate the 5-HT receptors of sensory neurons (Han and Dong, 2014). After depolarization of the 5-HT receptor, transient voltage receptor cation channel (TRP) family ion channels are activated, resulting in phosphorylation of extracellular regulated protein kinases (ERK). The skin sensory nerve fibers are excited and then itching signal is reduced (Yamaguchi et al., 1999; Zhao et al., 2014). At present, several animal models of acute itch treated by acupuncture have been studied. Electroacupuncture can effectively inhibit the degranulation of mast cells in mice with acute itching induced by compound 48/80 (Wang and Xu, 2014). Zhao et al. (2018) found that acupuncture can reduce the serum content of 5-HT in the mice with histamine-induced acute itching. Park H. J. et al. (2021) found that acupuncture can blockade the expression of 5-HT_2_ and 5-HT_7_ receptors in the skin of mice with serotonergic itch. Shao and Chen (2012) found that acupuncture can reduce the serum content of IgE and the concentration of 5-HT_2_A receptors in the skin of 5-HT-induced itching in rats. The 5-HT neurons in the central nervous system, involved in regulating the itch signal induced by various factors, play an important part of the itch signal transduction pathway (Akiyama et al., 2009, 2017). Related research found that mice lacking 5-HT or serotonergic neurons in the brainstem exhibit remarkably reduced scratching behavior (Zhao et al., 2014). In an animal model, acupuncture downregulated 5-HT neurons in the medulla oblongata, reducing excitability of nerve cells and inhibiting pruritus (Yan, 2017; Zhao et al., 2017). In humans, a recent fMRI result of experimental pruritus induced by histamine showed that the positive functional connectivity of putamen and the pMCC was associated with the antipruritic effects of acupuncture (Min et al., 2019).

## Mechanism of Acupuncture for Chronic Itch

Chronic itch is defined as pruritus lasting six or more weeks according to a recommendation by the International Forum for the Study of Itch (IFSI) (Ständer et al., 2007; Zhang et al., 2019). In heterogeneous forms of pruritus with dermatological disease accompanied by inflammation of the skin, persistent and continuous immune activation provoke a radical augmentation of histamine and mediators in the affected skin area, resulting in thickening and morphological changes of the epidermis, infiltration of a large number of immune cells, and chronic itching (Dong and Dong, 2018).

Closely related to the mechanism of acupuncture treatment of CP in the periphery is the cytokines released by immune cells, such as interleukin-4 (IL-4), interleukin-2 (IL-2), and interleukin-2 (IL-10). Increased IL-4 signaling was closely related to the development of pruritus with atopic dermatitis (Chan et al., 2001; Bogaczewicz et al., 2016). IL-4 directly promotes itch through the IL-4 receptor on itch-sensing neurons (Chiu et al., 2014; Usoskin et al., 2015). The activation of IL-4 receptor signal elevates the neuronal Ca^2+^ through the Janus kinase (JAK) pathway as well as both the transient receptor potential vanilloid V1 (TRPV1) and transient receptor potential ankyrin A1 (TRPA1) ion channels, resulting in activating the peripheral sensory nerve fibers and inducing chronic itch (Oetjen et al., 2017; Yosipovitch et al., 2018). By the regulation of cytokines, electroacupuncture reduces the generation of itching signals directly and inhibits the inflammatory response of the skin in the pruritus underlying dermatological disease. In humans, recent research results suggested that electroacupuncture reduced the level of IL-4 and IL-2 in the blood of patients with atopic dermatitis and increased the level of interferon γ (IFN-γ) (Deng, 2018). In patients with CP, electroacupuncture intensified the level of anti-inflammatory cytokine IL-10 in the serum and inhibited the content of inflammatory cytokine TNF-α, bringing about the reduction of inflammatory in the skin (Ni, 2017; Huang et al., 2020). However, serum IL-6 and interferon inducible protein (IP-10) may not be concerned with the antipruritic effect of acupuncture (Wang, 2018). In animals, a study found that electroacupuncture promoted the secretion of IFN-γ in the serum of mice with atopic dermatitis, while there was no sensible change about IL-4 (Jiang et al., 2016a).

Increasing evidence about central mechanism of CP suggested that microglial activity played a momentous role in the pathogenesis of CP evoked by 2,4-dinitrofluorobenzene (DNFB), compound 48/80, and 5′-guanidinonaltrindole (GNTI) (Liu et al., 2015; Ying et al., 2015; Zhang et al., 2015; Xu et al., 2020). Microglia exacerbate itch sensation by facilitating the phosphor-38 (p38) mitogen-activated protein kinase (MAPK) signaling pathway through chemokine CX3C receptor1 (CX3CR1) (Zhang et al., 2015). In addition, the suppression of the microglial-specific protein ionized calcium-binding adapter molecule 1 (lba1) in the spinal cord not only reduced the number of scratches in mice with atopic dermatitis, but repaired the afflicted skin area and improved inflammation of the skin (Torigoe et al., 2016). In Western blot analysis, electroacupuncture repressed the lba1 and phospho-p38 expression increased by s.c. injection of GNTI to the back of the neck in the spinal cord (Lin et al., 2016). A recent study showed a similar result in that manual acupuncture reduces cholestatic pruritus by alleviating spinal microglial activation (Lee et al., 2018).

Moreover, acupuncture in conjunction with the participation of multiple brain regions is one of the central mechanisms to antipruritics. In humans, the fMRI data outcome of patients with AD (Vitaly et al., 2014) showed that the activation of the right anterior insula, nucleus patella, putamen, globus pallidus, caudate nucleus, and nucleus accumbens was inhibited by electroacupuncture during the aggravation of itching. The activation of bilateral primary and right secondary somatosensory/motor cortex, middle frontal gyrus, cuneiform lobe, and left posterior cingulate cortex areas was reduced during the peak period of itching. The reduction of the intensity of itching was consistent with the inhibition of brain activation. Among these brain areas, they found that the reduction in putamen was closely correlated with the itch reduction following acupuncture.

## Mechanism of Acupuncture for Itch and Pain

Itch and pain are two different unpleasant somatic sensations with different behavioral manifestations. Pain causes the action of shrinking to avoid harmful stimuli, while itch causes the action of scratching to remove harmful substances invading the skin (McMahon and Koltzenburg, 1992). However, the sensations of itch and pain are also regarded as closely related. Itch can be suppressed by scratching and painful stimuli, whereas suppressed pain can elicit itch (Miyamoto and Patapoutian, 2011). One noteworthy reason for this appearance is that there is a broad crosstalk between pain- and itch-related mediators and/or receptors in the periphery and center, such as opioids and cannabinoids (Ikoma et al., 2006).

There are three opioid peptides, namely, β-endorphin (β-EP), enkephalin, and dynorphin (DYN), that are widely distributed in the periphery and the center. They modulate pain and itch by activating their respective opioid receptors. μ-Opioid system is itch-inducible, whereas the κ-opioid system is itch-suppressive (Kenya et al., 2017; Oeda et al., 2018). β-EP act as the agonist of μ opioid receptors to activate cognate morphine receptor-1D (MOR1D), which heterodimerizes with gastrin-releasing peptide receptor (GRPR, the itch-specific neurons) in the spinal cord to relay itch information (Liu et al., 2011). DYN, an endogenous κ-receptor agonist, is known to activate κ-opioid receptors (KORs) on GRPR-positive neurons and inhibit these neurons, resulting in attenuation of chemical itch (Mishra and Hoon, 2013; Yosipovitch et al., 2018; Chen et al., 2020). Moreover, DYN released by BhIhb5-1 neurons in the spinal dorsal horn is a neuromodulator to inhibit itch (Kardon et al., 2014).

An important target for acupuncture treatment of CP is β-EP, DYN, and μ and κ receptor systems. A paradoxical result was noteworthy in that acupuncture reduced the β-EP in the plasma of mice with CP, while it increased the expression of β-EP in the medulla oblongata (Yan, 2017). In the analgesic mechanism, β-EP plays a pivotal role in the central nervous system (Zhao, 2008). Therefore, it is inferred that when acupuncture stimulates mice with CP, the pain of acupuncture itself is also prominent, resulting in irritable upregulation of β-EP in central antinociception for analgesia. In addition, it had been found that electroacupuncture relieved pruritus in mice with atopic dermatitis *via* enhancing the synthesis and release of DYN at the spinal cord pruritus in rats to activate a large number of κ-receptors (Jung et al., 2014; Jiang et al., 2016b). Interestingly, different frequencies of electroacupuncture stimulated different opioid receptors. High frequency electroacupuncture (120 Hz) achieved the purpose of antipruritics by activating κ receptors. Nor-BNI-hydrochloride (k receptor antagonist) can effectively inhibit the antipruritic effect of 120-Hz electroacupuncture (Han et al., 2008). A stimulating finding showed that naloxone (the peripherally acting μ opioid receptor antagonist) partially reversed the antipruritic effect of low-frequency electroacupuncture (2 Hz), while the Nor-BNI-hydrochloride did not eliminate the antipruritic effect in mice with GNTI-induced pruritus (Chen et al., 2013).

Endogenous cannabinoids regulate pain and itch through specific membrane receptors, the cannabinoid receptors (Di Marzo and Petrocellis, 2006). It may be related to the physical increase of cannabinergic neurotransmitters after the activation of cannabinoid receptor 1 (CB_1_), which increases the pruritus threshold (Gingold and Bergasa, 2003). In animal models of chronic dry skin pruritus (Li, 2019), the expression of the CB_1_ in the midbrain tissue of mice was increased. Electroacupuncture reduced the scratching behavior through significantly downregulating the expression level of the cannabinoid receptor CB_1_. Intraperitoneal injection of CB_1_ receptor antagonist significantly reversed the effect of electroacupuncture. Li believed that the reason was that the antagonist antagonizes the CB_1_ receptors on both gamma aminobutyric acid (GABA) neurons and glutamatergic neurons, which may result in the domination of glutamatergic neurons to promote 5-HT release. Therefore, he implied that electroacupuncture participated in the central mechanism of acupuncture to relieve pruritus by regulating the CB_1_ receptor to inhibit the release of 5-HT. On the contrary, it has been suggested that antipruritic effects of cannabinoids are independent of descending inhibitory serotonergic pathways (Todurga et al., 2016).

## Challenges and Future Directions

**FIGURE 1:**
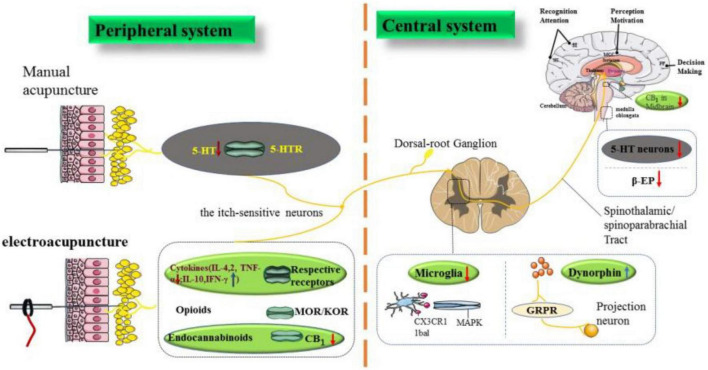
The neurobiological mechanism of acupuncture to relieve itching. The biomechanical stimulation signal of acupuncture can inhibit pruritus *via* acting on different levels of targets. In the periphery, manual acupuncture downregulates 5-HT (acute pruritus) and blocks its receptor expression. Electroacupuncture regulates cytokines and endocannabinoid receptor CB1 (chronic itch), and opioid peptide receptor is also one of the targets involved in acupuncture to relieve itching. In the center, acupuncture can regulate microglia and DNY in the spinal cord to treat chronic pruritus in animals. 5-HT neurons and β-EP in the medulla oblongata, and CB_1_ in the midbrain are also its targets. Putamen is a common target of acupuncture treatment for acute and chronic pruritus in humans. As shown in the figure, the red arrow represents the downregulation effect of acupuncture, while the blue arrow represents the upregulation effect of acupuncture. Among them, the purple font represents targets in humans, the yellow font represents both targets in humans and animals, and the rest represent animal research. The gray ellipses represent targets related to acute itch, the green ellipses represent targets related to chronic itch, and the others are both. SII, secondary somatosensory cortex; SI, primary somatosensory cortex; PF, prefrontal area.

The development of acupuncture represents a prime example of non-pharmacologic treatment. The currently available acupuncture therapy has shown high response rates and long-term remissions in patients with acute or chronic itch with minimal side effect. In this review, the authors provide an overview of the effect in acupuncture recipients and of the method and acupoint in acupuncture management (Figure 1). Furthermore, relieving itching of acute and chronic mechanisms by acupuncture is discussed.

The neurobiological mechanism of acupuncture to antipruritics that has made some progress is less in-depth, compared with the already clear mechanism of pruritus. There is a phenomenon of circadian variation of pruritus, and pruritus is usually much severe at night (Millington et al., 2018; Weisshaar et al., 2019). There are many research evidence on the adjustment effect of acupuncture on the circadian rhythm (Kim et al., 2012). For instance, electroacupuncture at different hours produced different effects on leucine-enkephalin contents in rat medulla oblongata regions (Wang and Wang, 1989). It is worthy to explore the mechanism in this area. Chronic pruritus is also often associated with psychopathology, such as anxiety and depression (Gupta and Gupta, 1999). Acupuncture may improve itching by treating related mental illnesses. Unfortunately, research has not yet been conducted.

In conclusion, further application of acupuncture therapy for pruritus will require a better understanding of the peripheral and central, molecular, and cellular mechanisms. Using *in vivo* calcium imaging, modern genetics, and imaging tools and other technologies, we can expand our understanding of the related circuits in the spinal cord and brain for acupuncture to relieve itching. In addition, cytokines, endocannabinoids and the CB1 receptor, and opioids and their receptors are important targets of acupuncture antipruritic mechanisms worth paying attention to.

## Author Contributions

YT and SC performed document retrieval and manuscript writing. XW designed the protocols. JW and YJ performed the literature screening. JW contributed to making Tables 1, 2. QL, HY, and SX performed the data extraction and data analysis. YY and QY performed charts. QY has contributed to the data and make of figure for the work. All authors contributed to the article and approved the submitted version.

## Conflict of Interest

The authors declare that the research was conducted in the absence of any commercial or financial relationships that could be construed as a potential conflict of interest.

## Publisher’s Note

All claims expressed in this article are solely those of the authors and do not necessarily represent those of their affiliated organizations, or those of the publisher, the editors and the reviewers. Any product that may be evaluated in this article, or claim that may be made by its manufacturer, is not guaranteed or endorsed by the publisher.
